# Application Effect of the China Association For Disaster and Emergency Rescue Medicine - Cardiopulmonary Resuscitation and Automatic Extracorporeal Defibrillation (CADERM-CPR·D) Training in Medical Teaching

**DOI:** 10.7759/cureus.52412

**Published:** 2024-01-16

**Authors:** Yaoliang Zhou, Hujie Zhang, Chuyu Xie, Li Xu, Xiaoyu Huang

**Affiliations:** 1 Emergency and Disaster Medicine Center, The Seventh Affiliated Hospital of Sun Yat-sen University, Shenzhen, CHN

**Keywords:** training, application effect, automatic extracorporeal defibrillation, cardiac arrest, cardiopulmomary resuscitation, case guidance

## Abstract

Objective

In China, the penetration rate of cardiopulmonary resuscitation training is not high and the effect of traditional teaching methods is not good. In this study, the case-guided cardiopulmonary resuscitation training mode was introduced to provide cardiopulmonary resuscitation training to medical students with a certain medical background, using the 2018 technical specifications for cardiopulmonary resuscitation and automatic extracorporeal defibrillation of the China Association for Disaster and Emergency Rescue Medicine. Compared with traditional teaching methods, the application effect of this training method in clinical probation teaching was analyzed.

Methods

120 medical students with a certain medical background were randomly divided into the experimental group and the control group, with 60 students in each group. The knowledge, skills, and attitude of the subjects were assessed by questionnaire survey.

Results

A total of 120 students were included in the study and randomly divided into an experimental group and a control group. The test scores of knowledge, skill, and attitude in the experimental group (38.40±2.775, 19.07±1.118, 14.92±0.962) were significantly higher than those in the control group (32.47±3.615, 14.65±1.338, 12.68±0.930)(P<0.05).

Conclusion

Case-guided cardiopulmonary resuscitation training of the China Association for Disaster and Emergency Rescue Medicine specifications can improve medical students' knowledge and skills of cardiopulmonary resuscitation, enhance their confidence in treatment, and can be further applied in medical teaching.

## Introduction

Cardiac arrest (CA) is an emergency situation [1], which seriously endangers human health and requires timely cardiopulmonary resuscitation (CPR) to maintain life. CA patients should be given high-quality external chest compression and external defibrillation. Early initiation of basic life support is the most effective means to restore autonomous circulation [2-3]. However, the penetration rate of CPR training in China is not high, especially the resuscitation rate of out-of-hospital cardiac arrest is far lower than the international standard [4]. This is mainly due to inadequate CPR training and low public penetration [5]. College students have strong self-learning ability and thinking ability. By improving their CPR skills, they can not only provide timely rescue in emergencies but also popularize the knowledge they have learned to the people around them, so as to improve the public's awareness and mastery of CPR. Therefore, more efforts should be made to train college students in CPR skills, and college students' CPR skills should be improved by setting up special courses and providing practical operation opportunities [6]. Only in this way can we increase the prevalence of CPR, improve the resuscitation rate of cardiac arrest patients, and contribute to public health.

China Association for Disaster and Emergency Rescue Medicine (CADERM) was founded in 2009. It is an academic and non-profit social organization composed of medical workers engaged in emergency and first aid and rescue workers in social-related fields. In 2018, CADERM launched the technical specifications for Cardiopulmonary Resuscitation and Automatic Extracorporeal Defibrillation (CPR·D) [7] with the goal of improving the survival rate of patients with cardiac arrest. The course adopts the situational simulation teaching form of "case guidance + watching while practicing + case simulation assessment", and the training is carried out in groups. This paper takes the medical students as the research object, compares the training mode with the traditional teaching method, and discusses the application effect of the training mode in the practical teaching of medical students.

## Materials and methods

The ethics of this study have been approved by the Medical Ethics Committee of the Seventh Affiliated Hospital of Sun Yat-sen University, and all procedures involving human participants in this study are in accordance with the ethical standards of the Institutional Research Committee and comply with the 1964 Declaration of Helsinki and its later amendments or similar ethical standards.

Training targets

We recruited 120 clinical medical students of Class 2019 from the School of Medicine of Sun Yat-sen University who will be clinical interns in our hospital from March 2023 to May 2023 and had never participated in CPR training before. They were randomly divided into two groups by computer-randomized method: 60 people in the experimental group received CPR training according to the CADERM-CPR·D teaching method; the control group of 60 people was trained by the traditional teaching method [8-9].

Qualifications of tutors

The Chinese Medical Rescue Association has formulated a set of standardized teacher training procedures, and teachers participating in the teaching must be trained and assessed in accordance with the association's standards to obtain the qualification of CPR first aid instructor. Most of these teachers come from the Emergency and Disaster Medicine Center of the Seventh Affiliated Hospital of Sun Yat-sen University, Intensive Care Medicine department, the cardiovascular center, and other clinicians and nurses with rich experience in first aid. They have rich practical experience and theoretical knowledge and can better teach. In addition, senior tutors are arranged for teaching supervision from time to time to ensure the quality of teaching. At the same time, according to the training management requirements of the Chinese Medical Rescue Association, the tutor needs to participate in the re-training after every 3 years in order to renew the tutor qualification.

Training methods

Experimental Group

In group training, each group is assigned CADERM-awarded first aid instructor qualified instructors. The student-to-tutor ratio is not higher than 6:1. Training was done using "case guidance + practice while watching videos + case simulation assessment" scenario simulation form of training in stages, including adult, child, infant CPR rescue, Automatic Extracorporeal Defibrillation (AED) use, and other content [1]. After the completion of training, the students' knowledge, skills, and attitudes were evaluated.

Control Group

According to the traditional teaching method, the theoretical knowledge of CPR is taught in the classroom, and CADERM grants first aid instructors qualified to demonstrate the operation first, and the students then practice the training, with each instructor instructing a maximum of six students. After the completion of training, the students' knowledge, skills, and attitudes were evaluated.

The training duration of each of the two groups was 4 hours, the training was done only once.

Questionnaire

The Cardiopulmonary Resuscitation Training Questionnaire was designed by referring to relevant literature [10-11] and combining it with the 2018 Technical Specifications for On-site Cardiopulmonary Resuscitation and Automatic External Cardiac Defibrillation (CPR·D) [1] of the Chinese Medical Rescue Association. The questionnaire included the following contents:

General Information

Investigate the gender and cultural background of the trainees.

CPR-Related Knowledge, Skills, Attitude Assessment Questionnaire

Knowledge part: Including basic knowledge of CPR, chest compression, artificial ventilation, electrical defibrillation, and other content; a total of 10 single-choice questions; correct answers to each question were awarded 4 points, with a full score of 40 points.

Skills part: Including the recognition of cardiac arrest, external chest compression, open airway, mouth-to-mouth resuscitation, and mastery of electrical defibrillation - a total of five items are included. The Likert 4-level scoring method is used to score - 1 point for "totally unaware", 2 points for "less aware", 3 points for "basically aware", and 4 points for "fully aware" [12], with a full score of 20.

Attitude part: A total of four items, using a Likert 4-level scoring method - "none at all" counts for 1 point, "generally not" counts for 2 points, "some or have" counts for 3 points, "very" counts for 4 points, with a full score of 16 points.

The higher the score, the better the grasp of cardiopulmonary resuscitation and the stronger the first aid intention.

Effect evaluation

After the training, the students in the two groups were given questionnaires on CPR to investigate the effect. A total of 120 questionnaires were given out, and 120 were effectively recovered, with a recovery rate of 100% (n=120). After the survey data were input and reviewed, SPSS 26.0 software (IBM Corp., Armonk, USA) was used for data analysis and processing. The measurement data were presented as mean value and standard deviation (x̄±s), and the counting data were presented as frequency and frequency. Paired T-test was used, and P < 0.05 was considered statistically significant.

## Results

General information questionnaire results

The 120 respondents were all grade 2019 clinical medical students of the School of Medicine of Sun Yat-sen University who were clinical interns in our hospital from March 2023 to May 2023. There were 31 female students and 29 male students in the experimental group. The control group consisted of 33 girls and 27 boys.

Results of the CPR-related knowledge, skills, and attitude assessment questionnaire

After the training, the students in the two groups received the knowledge, skills, and attitude survey, and the scores of knowledge, skills, and attitude were calculated according to the scoring method developed in the study. The scores and comparisons of the students in the experimental group and the control group are shown in Table 1 and Table 2. Using the statistical method of paired T-test, as can be seen from Table 1, the scores of the knowledge part are: the experimental group (38.40±2.775) points, the control group (32.47±3.615) points, and the difference is statistically significant (P < 0.001). The scores of the skill part are: the experimental group (19.07±1.118) points, the control group (14.65±1.338) points, and the difference was statistically significant (P < 0.001). The scores of the attitude part are: the experimental group (14.92±0.962) points, the control group (12.68±0.930) points, and the difference was statistically significant (P < 0.001). In addition, it can be seen from Table 2 that the experimental group scored higher than the control group in skills such as recognition of respiratory and cardiac arrest, external chest compression, open airway, mouth-to-mouth resuscitation, and electrical defibrillation, with statistical significance (P < 0.001). The scores of the experimental group on the importance of CPR, the necessity of training, the willingness to rescue, and the confidence to save were higher than those of the control group, the difference was statistically significant (P < 0.01).

**Table 1:** Assessment scores and comparison between two groups of students SD: standard deviation

Characteristic	Experimental group (n=60)	Control group (n=60)	P-value
Knowledge, Points, Mean (SD)	38.40 (2.775)	32.47 (3.615)	＜0.001
Skill, Points, Mean (SD)	19.07 (1.118)	14.65 (1.338)	＜0.001
Attitude, Points, Mean (SD)	14.92 (0.962)	12.68 (0.930)	＜0.001

**Table 2:** Comparison of scores of skills and first aid willingness after training between two groups of students CAR: cardiac arrest recognition; CC: chest compression; OA: open airway; MTMB: mouth-to-mouth breathing; ED: electric defibrillation; CPRS: cardiopulmomary resuscitation significance; TN: training necessity; WTHS: willingness to help strangers; CIT: confidence in treatment; SD: standard deviation

Characteristic	Experimental group (n=60)	Control group (n=60)	P-value
CAR, points, mean (SD)	3.82 (0.390)	3.03 (0.486)	＜0.001
CC, points, mean (SD)	3.85 (0.404)	2.95 (0.502)	＜0.001
OA, points, mean (SD)	3.80 (0.403)	2.85 (0.547)	＜0.001
MTMB, points, mean (SD)	3.77 (0.465)	2.83 (0.557)	＜0.001
ED, points, mean (SD)	3.83 (0.376)	2.98 (0.537)	＜0.001
CPRS, points, mean (SD)	4.00 (0.000)	3.62 (0.490)	＜0.001
TN, points, mean (SD)	3.87 (0.343)	3.63 (0.520)	＜0.01
WTHS, points, mean (SD)	3.48 (0.701)	2.98 (0.390)	＜0.001
CIT, points, mean (SD)	3.57 (0.533)	2.45 (0.565)	＜0.001

## Discussion

Importance of cardiopulmonary resuscitation

Cardiac arrest, with its high incidence and fatality rate [13-14], low survival rate, and unclear upward trend [15-16], is one of the major global public health problems. Cardiopulmonary resuscitation (CPR) is an effective means to save the lives of patients with cardiac arrest [17]. Studies have shown that if effective CPR is performed within 3 to 5 minutes after the initial occurrence of cardiac arrest, the survival rate is higher, up to 49% to 75%. However, for every 1-minute delay in CPR, the survival rate of patients decreases by 7% to 10%[18-19]. The improvement of the survival rate of patients depends on timely and high-quality intervention in all links of the survival chain, including timely activation of the emergency system [20], increasing the CPR participation rate of witnesses [21], and shortening the defibrillation time [22]. External chest compression is the key to CPR. The content includes the best position and/or point of the hand during compressions, and the optimal depth and frequency of external chest compressions [23-27], which determines the quality of the entire CPR [28] and requires repeated operation practice.

The importance of popularizing CPR training for college students

College students are an important force for the dissemination of cardiopulmonary resuscitation knowledge, and they have a strong learning ability. However, the awareness and mastery of CPR among Chinese college students still need to be improved [29]. Therefore, effective measures should be taken, such as holding regular special lectures, providing practical operation opportunities, and using WeChat, Weibo, Douyin, and other social-media to spread knowledge and skills of cardiopulmonary resuscitation, so as to improve college students' cardiopulmonary resuscitation skills. At the same time, we should encourage college students to actively learn and spread the knowledge of CPR, apply it to real life, and improve the public's awareness and mastery of CPR. As future medical workers, medical students should undertake the responsibility of saving lives and healing the wounded. In addition to solid professional theoretical knowledge and clinical practice skills, they should also have professional self-confidence, pressure resistance, social responsibility, and other humanistic qualities [30]. According to this investigation, the scores of the students' willingness to rescue after the training of the two groups are both high, which indicates that after learning relevant undergraduate medical knowledge, most of the students have the consciousness of "saving lives and healing the wounded" and are willing to apply the medical knowledge they have learned to practice. However, there are obvious differences between the two groups of students' confidence in saving lives. Students with CADERM-CPR·D training mode have higher rescue confidence than traditional teaching. This training mode can improve rescue confidence because of its situational simulation teaching method so that students can practice repeatedly on the simulation equipment, find and correct wrong operations in time, and constantly improve their knowledge, skills, and self-confidence.

Application effect of CADERM-CPR·D training method in medical teaching

The scenario simulation training of "case guidance + practice while watching video + case simulation assessment" requires students to complete one or a series of tasks according to the work requirements by setting realistic first aid scenes, from which to exercise or examine their first aid ability and level, which has intuitive, graphic, and vivid characteristics. Compared with traditional teaching methods, students are allowed to experience the rescue atmosphere in real cases, increase their initiative and participation, and combine basic knowledge, clinical thinking, and clinical operation with video to deepen students' understanding and memory of the training content.

There are still some limitations in this study. The number of participants is relatively small and their age is relatively similar. The sustainability and effectiveness of the training effect need to be further studied in the future.

## Conclusions

In this study, students using the CADERM-CPR·D training method had significantly higher scores in cardiopulmonary resuscitation knowledge, skills, and attitudes than those using traditional training methods. Students not only had high learning enthusiasm and good results, but they also had significantly enhanced confidence in treatment. Therefore, the CADERM-CPR·D training model can improve the first aid skills of medical students and can be further applied in clinical medical practice teaching, which is worthy of widespread promotion.
